# In Vivo-Acquired Resistance to Daptomycin during Methicillin-Resistant *Staphylococcus aureus* Bacteremia

**DOI:** 10.3390/antibiotics12121647

**Published:** 2023-11-22

**Authors:** Adeline Boutet-Dubois, Chloé Magnan, Alexi Lienard, Cassandra Pouget, Flavien Bouchet, Hélène Marchandin, Romaric Larcher, Jean-Philippe Lavigne, Alix Pantel

**Affiliations:** 1 VBIC, INSERM U1047, Department of Microbiology and Hospital Hygiene, University of Montpellier, CHU Nîmes, 30029 Nîmes, France; adeline.dubois@chu-nimes.fr (A.B.-D.); chloe.magnan@chu-nimes.fr (C.M.); cassandra.pouget@chu-nimes.fr (C.P.); alix.pantel@chu-nimes.fr (A.P.); 2 Laboratory of Medical Biology, CH Bassin de Thau, 34207 Sète, France; alienard@ch-bassindethau.fr; 3 Department of Internal Medicine-Infectiology, CH Bassin de Thau, 34207 Sète, France; fbouchet@ch-bassindethau.fr; 4 HydroSciences Montpellier, Department of Microbiology and Hospital Hygiene, University of Montpellier, CNRS, IRD, CHU Nîmes, 30029 Nîmes, France; helene.marchandin@umontpellier.fr; 5 Department of Infectious Diseases, CHU Nîmes, 30029 Nîmes, France; romaric.larcher@chu-nimes.fr

**Keywords:** daptomycin resistance, *mprF* mutations, *cls* mutation, Methicillin-resistant *Staphylococcus aureus*, whole genome sequencing

## Abstract

Daptomycin (DAP) represents an interesting alternative to treat methicillin-resistant *Staphylococcus aureus* (MRSA) infections. Different mechanisms of DAP resistance have been described; however, in vivo-acquired resistance is uncharacterized. This study described the phenotypic and genotypic evolution of MRSA strains that became resistant to DAP in two unrelated patients with bacteremia under DAP treatment, in two hospitals in the South of France. DAP MICs were determined using broth microdilution method on the pairs of isogenic (DAP-S/DAP-R) *S. aureus* isolated from bloodstream cultures. Whole genome sequencing was carried out using Illumina MiSeq Sequencing system. The two cases revealed DAP-R acquisition by MRSA strains within three weeks in patients treated by DAP. The isolates belonged to the widespread ST5 (patient A) and ST8 (patient B) lineages and were of *spa*-type t777 and t622, respectively. SNP analysis comparing each DAP-S/DAP-R pair confirmed that the isolates were isogenic. The causative mutations were identified in MprF (Multiple peptide resistance Factor) protein: L826F (Patient A) and S295L (Patient B), and in Cls protein: R228H (Patient B). These proteins encoded both proteins of the lipid biosynthetic enzymes. The resistance to DAP is particularly poorly described whereas DAP is highly prescribed to treat MRSA. Our study highlights the non-systematic cross-resistance between DAP and glycopeptides and the importance of monitoring DAP MIC in persistent MRSA bacteremia.

## 1. Introduction

Methicillin-resistant *Staphylococcus aureus* (MRSA) infections represent a major health problem, leading to high morbidity and mortality worldwide [1,2]. MRSA strains are resistant to nearly all β-lactams and several other classes of antibiotics such as aminoglycosides, fluoroquinolones, and tetracyclines, restricting the antimicrobial chemotherapy possibilities against those infections. Glycopeptides have long been considered the first-line therapy for severe MRSA infections [1]. However, new antibiotic solutions have emerged to treat infections due to intermediate or resistant vancomycin *S. aureus*. Daptomycin (DAP) represents an interesting alternative treatment of MRSA [1]. This antibiotic is a calcium-dependent lipopeptide that is increasingly used for the treatment of MRSA bloodstream infections due to higher treatment success rate [3] and reduced risk of mortality compared to vancomycin (VAN) [4]. 

DAP has been isolated from *Streptomyces roseosporus* [5]. This antibiotic has been approved for use and continues to be considered a mainstay of anti-MRSA treatment [6]. It possesses a cyclic peptide core linked to a fatty acyl chain [7]. Associated with calcium, this DAP-Ca^2+^ complex forms tetramers within the outer leaflet and constitute an amphipathic complex that facilitates oligomerization [8]. Finally, the DAP-Ca^2+^ complex forms micelles, which are able to insert into the cell wall of bacteria and bind to phosphatidylglycerol [9]. Thus, these micelles can disrupt the cytoplasmic membrane of Gram-positive bacteria, involving a depolarization, permeabilization, and ion leakage of the bacterium [10,11,12]. Moreover, the DAP-Ca^2+^ complex masks the negative charge of lipopeptides increasing its affinity for one of the major anionic phospholipids present in the Gram-positive membranes [13]. Multiple DAP mechanisms of action have been published such as membrane permeabilization, inhibition of cell wall synthesis, physical alteration of membrane curvature and fluidity, and disruption of the peptidoglycan synthesis machinery [10,14,15,16,17]. Finally, this antibiotic harbors bactericidal and non-bacteriolytic properties. Based on pharmacokinetic studies, DAP exhibits concentration-dependent killing [18]. 

DAP-resistance is a rare event. Several explanations have been provided. This resistance is mainly associated with the positively-charged membrane of the cell due to alanylation of teichoic acids and the lysinylation of phosphatidylglycerol in cell membrane, repelling the antibiotic. These modifications change the membrane fluidity and reduce the affinity of cationic antimicrobial peptides [19]. In strains not susceptible to DAP, different studies have documented the presence of mutations in various genes encoding enzymes involved in the phospholipid synthesis and metabolism: *mprF* (multiple peptide resistance Factor), *cls* (cardiolipin synthase)*,* and *pgsA* (phospholipid metabolism). Mutations have also been described into two component systems regulating cell membrane stress and permeability, *walk/walR* (also known as *yycF/yycG*) and *vraS/vraR*. Finally, the upregulation of *dltABCD* transcription contributed to enhance the surface positive charge by teichoic acid D-alanination, as observed with *mprF* mutations [20,21,22,23]. However, the in vivo-acquired resistance is rarely described [24,25,26] and some associations between the resistance to VAN and the decreased susceptibility to DAP have been reported [7]. Here, we described the phenotypic and genotypic evolution of MRSA strains that became resistant to DAP in two unrelated patients with bacteremia under DAP treatment only, in two hospitals in the South of France.

## 2. Results

### 2.1. Patient A

The patient was an 81-year-old man followed in Nîmes University Hospital (France) for a lung adenocarcinoma discovered in 2012. The patient had developed cerebral metastasis, and after several anti-cancer chemotherapies and interventions, immunotherapy was initiated in February 2020. On April 2021, the patient presented to the Emergency Department at Nîmes University Hospital with local pain next to his central vascular catheter (Port-a-Cath), implanted on three days after. The home nurse also reported a purulent flow for several days. The patient was apyretic, but bloodstream cultures were performed. They incubated in the Bactec FX system (Becton Dickinson, Franklin Lakes, NJ, USA) and were positive 12 h after collection, with Gram-positive cocci observed on Gram staining. The patient was hospitalized in the Pneumology Unit. The Port-a-Cath was removed after two weeks. A probabilistic intravenous antimicrobial therapy combining DAP with piperacillin/tazobactam (TZP) was initiated. The bacterial culture of the device identified a DAP-susceptible (DAP-S) MRSA (MIC of 0.5 mg/L). TZP was stopped three days after and DAP monotherapy was continued at 10 mg/kg/24 h. Despite correct antimicrobial treatment, bloodstream cultures remained positive for *S. aureus.* After two weeks, an antimicrobial susceptibility testing identified DAP-resistant (DAP-R) MRSA isolates (MIC of 2 mg/L). Antimicrobial therapy was switched to intravenous ceftaroline. The patient died two weeks after in palliative care due to complications of his cancer.

### 2.2. Patient B

The patient was a 69-year-old man diagnosed with Parkinson disease in 2017. He was first hospitalized in Sète General Hospital (France) in October 2020 for surgical treatment of colorectal anastomosis. In June 2021, the patient was re-hospitalized for restoration of intestinal continuity. This stay was complicated with stercoral peritonitis secondary to suture breakage 5 days after surgery. The intraoperative microbiological cultures showed *Escherichia coli*, *Proteus mirabilis, Enterococcus faecalis,* and *Enterococcus avium.* An antimicrobial therapy associating TZP to fluconazole was initiated. Two weeks after, an abdominal CT scan highlighted a sub-diaphragmatic collection that was radiologically drained. Empiric intravenous antimicrobial therapy with TZP was prescribed. *E. coli*, *Enterococcus faecalis*, *P. mirabilis,* and *Staphylococcus capitis* were isolated. Intravenous antimicrobial treatment was switched to meropenem at 6 g/24 h and DAP at 10 mg/kg/24 h for three weeks. At end of July, antimicrobial therapy was stopped. At the beginning of September 2021, the patient was febrile (39 °C) after removal of the PICC-line and subcutaneous catheter. Bloodstream cultures identified a DAP-S MRSA (MIC of 0.5 mg/L). Intravenous antimicrobial therapy with DAP at 10 mg/kg/24 h was initiated for 14 days. Three weeks after, the patient was febrile again with CRP at 176 mg/L. Bloodstream cultures revealed DAP-R MRSA (MIC of 2 mg/L). Antimicrobial therapy was switched to intravenous ceftaroline and clindamycin intravenous association. There was no relapse, and the patient was transferred to a rehabilitation center. 

### 2.3. Description of the Two DAP-R MRSA Isolates

The main characteristics of the pairs of isogenic (DAP-S/DAP-R) *S. aureus* strains are described in Table 1.

The DAP-S (MIC of 0.5 mg/L) MRSA strain isolated from patient A was susceptible to glycopeptides (VAN, TEI and DAL MICs of 1, 0.5 and 0.06 mg/L, respectively) and to advanced generation cephalosporins (CPT and CBP MICs 0.25 and 1 mg/L, respectively). The isolate was resistant to β-lactams (penicillin G and oxacillin) and fluoroquinolones (ofloxacin). After 21 days of exposure to DAP, the strain was resistant to this antibiotic (MIC of 2 mg/L) but no cross-resistance to glycopeptides was observed (VAN, TEI and DAL MICs of 1, 1 and 0.06 mg/L, respectively).

The DAP-S MRSA (MIC of 0.5 mg/L) isolated from patient B was also susceptible to glycopeptides (VAN, TEI and DAL MICs of 1, 0.5 and 0.06 mg/L, respectively) and to CPT and CBP (MIC of 0.38 and 1.5 mg/L, respectively). The isolate was resistant to rifampicin and fluoroquinolones. After 22-days exposure to DAP, a MRSA isolate was detected also resistant to DAP (MIC of 2 mg/L).

### 2.4. Whole Genome Analysis of the DAP-S/DAP-R Isolates

The wgMLST analysis showed that MRSA isolates belonged to the widespread ST5 (patient A) and ST8 (patient B) lineages and were of *spa*-type t777 and t622, respectively (Table 1). Genome assembly generated median of 2,817,995 bp in size for isolates from patient A and 2,881,993 bp for isolates from patient B, corresponding to median coverage >97% and 0.030% in gap ratio. Whole genome annotation of *S. aureus* genomes from patient A predicted a median of 32.67% of CG content for A_DAP-S and A_DAP-R. The median coding ratios were 99.88% for both isolates including a median of 2530 and of 2529 coding sequences (CDS), and both 61 tRNA and 12 rRNA, respectively. The annotation for isolates from patient B predicted a median of 32.63% of CG content for B_DAP-S and B_DAP-R. The median coding ratios were 99.85% and 99.81%, including a median of 2617 and 2612 CDS, and both 61 tRNA and 10 rRNA for the two isolates, respectively. SNP analysis comparing each pair of DAP-S/DAP-R isolates from each patient confirmed that the isolates were isogenic. For both patients, we observed only one SNP difference between each isolate across the entire genome.

### 2.5. Resistome Profiling of the DAP-S/DAP-R Isolates

The four *S. aureus* isolates were predicted in silico to be resistant to penicillin G, methicillin by the presence of *blaZ* and *mecA* genes. The resistance to ofloxacin was provided by point mutations in DNA gyrase subunit A- (*gyrA*) and topoisomerase IV subunit C (*parC*)- encoding genes harbored in all isolates conferring resistance to fluoroquinolones. Isolates from patient B presented classic mutation (D471E) usually predicted in the *rpoB* gene and involved in rifampicin resistance. Finally, the four isolates had *mepA* gene, which encoded efflux pumps and the two isolates from patient B harbored point mutations in *murA* gene, which is responsible for the early rate-limiting step in cell wall synthesis. 

### 2.6. Virulome of the DAP-S/DAP-R Isolates 

For the virulome profile of the *S. aureus* isolated from patient A, the whole genome analysis showed that all strains carried enterotoxins- (*sea*, *sed*, *sei, sej, sem, sen, seo,* and *ser*), leukocidins- (*lukF*, *lukS*, *lukH, lukD*, *lukE* and *lukY*), adhesion factors- (belonging to the microbial surface components recognizing adhesive matrix molecules: *clfA, clfB*, *fnbA*, *fnbB*, *fib*, *map*, *eno*, *efb*, *ebpS*, *sdrC*, and *sdrE*), and hemolysins- (*hla*, *hlb*, *hld*, *hlgA, hlgB* and *hlgC*) encoding genes. The isolates also harbored the capsule- (*cap8*), the clipase- (*clpB, clpC, clpP, clpX*), the biofilm formation- (*ica* operon), the protease- (*splABCDF*), and the immune evasion cluster- (*sea, sak, chp* and *scn*) encoding genes. Finally, the isolates presented the specialized secretion system Ess (ESAT-6 secretion system) with the *ess* locus, a cluster of eight genes (e.g., *esxAB, essABC, esaABC*). However, these isolates harbored neither exfoliative toxin-encoding genes (*eta*, *etb*, *etd*), nor epithelial cell differentiation inhibitors (*edinA*, *edinB*, and *edinC*), the toxic shock syndrome toxin- (*tst*), and *lukF/S-PV* encoding genes.

For the virulome profile of the *S. aureus* isolated from patient B, the genomic content was similar to isolates from patient A. The differences were the absence of *chp*, the content of enterotoxins-encoding genes (*sea, sed, sej, ser* and *set*) and the presence of the adhesin- (*map*) and the von Willebrand factor-binding protein- (*vWbp*) encoding genes. 

### 2.7. Genetic Basis of Resistance to DAP

**Figure 1:**
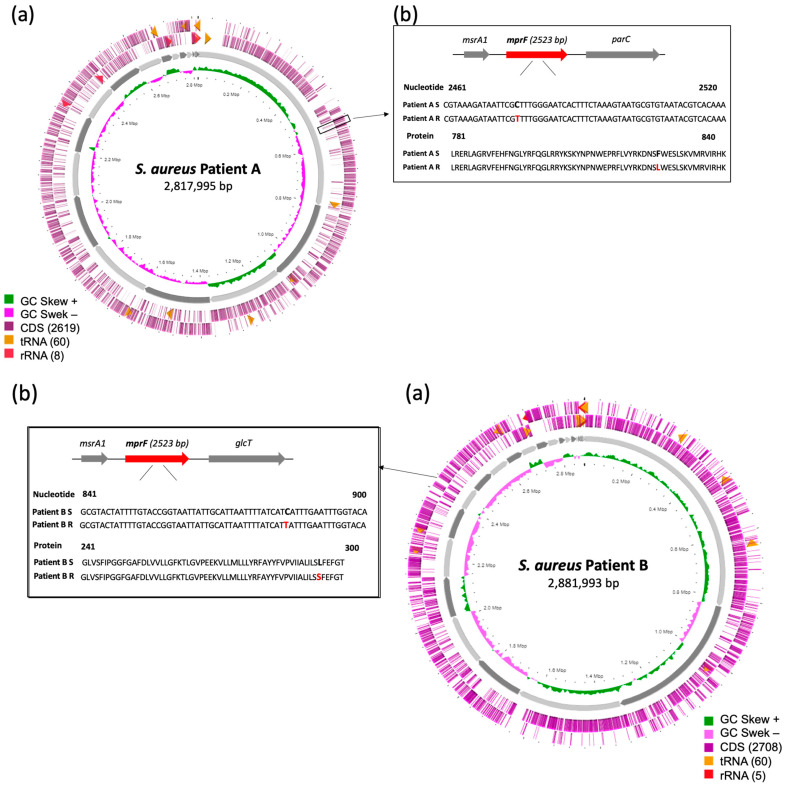
The *mprF* genomic landscape. (**a**) Circular genome representation of patient A and patient B MRSA strains using BRIG software. The inner ring illustrates the GC skew. The outer two rings represent coding sequences (CDS), tRNAs and rRNAs on the reverse and forward strands, respectively. A black box is included to highlight the *mprF* gene. (**b**) Genomic localization of *mprF*. Nucleotide and protein alignments of DAP-susceptible/DAP-resistant isolates from patients A and B, and the mutations detected.

A focus on genes involved in DAP resistance was performed. For Patient A, one allelic difference was detected between the two isolates, consisting in one mutation located in MprF protein (L826F) (Figure 1). For Patient B, two allelic differences were noted between the two isolates corresponding to another mutation in MprF protein (S295L) and another one in Cls protein (R228H).

## 3. Discussion

DAP resistance remains a rare event. Main reports described clinical cases following glycopeptide and DAP therapy [24,27]. In fact, prior exposure to glycopeptides are highlighted as risk factors for decreased susceptibility to DAP [24,28]. These antibiotics induced changes of the bacterial cell wall [28,29]. In this study, we provided two cases of emerging DAP resistance in MRSA during bacteremia, resulting in treatment failure of only DAP and consequently long-term bacteremia. One of the two patients died when adapted treatment was prescribed, but the link between mortality and the presence of DAP-R strains could not be deduced because this patient approached his end of life. Interestingly, no DAP resistance development was preceded by a modification of VAN susceptibility and no other co-resistance was noted as previously observed [24]. Moreover, it could be observed that DAP was administrated at a well-established dose. However, we cannot evaluate the blood concentration of DAP. There is evidence that a subtherapeutic concentration of this antibiotic favors the development of resistance as previously noted [30].

Globally, the studies on DAP resistance mainly focus on the interaction between the cell membrane and DAP–Ca^2+^ complex [19,20,21,22,23,24,25,26]. The two pairs of isogenic strains showed distinct mutations in the *mprF* gene. The transmembrane MprF protein is responsible for the lysinylation of cell membrane phospholipids and their translocation to the outer leaflet of the cell membrane [31]. This process decreases the negative cell surface charge, leading to the electrostatic repulsion of the cationic anti-microbial peptides such as DAP. Furthermore, this rearrangement has been described as a virulence factor and is widespread in Gram-positive and Gram-negative bacteria [31]. The DAP-R emergence has a fitness cost on the MRSA, with changes in cell wall thickness and cell membrane potential [32]. The MprF protein consists of an N-terminal transmembrane flippase domain, C-terminal catalytic synthase domain, and a central bifunctional domain. The changes in amino acid sequences leading to DAP-R *S. aureus* have been identified in all domains of MprF [33]. S295L mutation was associated with a change in the central bifunctional domain while L826F was related to a change in synthase domain [34]. Cross-resistance to VAN was previously described as associated with *mprF*-mediated DAP-R [35]. Moreover, Capone et al. observed a link between the emergence of in vivo DAP-R *S. aureus* and the glycopeptide therapy [24]. Neither phenomena were observed in our cases; no patients received glycopeptides. 

Interestingly, the strain from patient B presented a mutated Cls protein (R228H). A relation between *cls* and DAP resistance has been previously described but exclusively in *Enterococcus* [36,37]. Zhang et al. showed that *cls* gene encoded a cardiolipin synthase, which catalyzed the production of cardiolipin from two molecules of phosphatidylglycerol. The authors suggested that the increase in the concentration of cardiolipin in cell membranes can divert more DAP from its target septum to other sites, thereby improving DAP resistance [9]. The *cls* mutations could modify the distribution of cardiolipins in cell membrane, involving a reduction of these lipids on the cell surface and decreasing the negative charges of membrane cells leading to a reduced adhesion to DAP [37]. We could note that the mutation detected in Cls protein had never been previously described in *Enterococcus* sp. This mutation affected one of phospholipase D-like domains that could alter the catalytic activity of cardiolipin synthase [37]. Investigation on the clear role of the Cls mutation detected in our study and its addition to MprF mutation could be provided in future analysis. 

## 4. Materials and Methods

### 4.1. Bacterial Identification and Antibiotic Susceptibility Testing

Isolates were identified by mass spectrometry using Vitek^®^ MS system (bioMérieux, Marcy L’Etoile, France) and stored in cryotubes at −80 °C. Antimicrobial susceptibility testing (Penicillin G 1U, cefoxitin 30 μg, erythromycin 15 μg, clindamycin 2 μg, quinupristin-dalfopristin 15 μg, kanamycin 30 μg, tobramycin 10 μg, gentamicin 10 μg, minocycline 30 μg, ofloxacin 5 μg, fusidic acid 10 μg, fosfomycin 200 μg, rifampicin 5 μg, cotrimoxazole 25 μg, linezolid 10 μg) of these isolates was performed by disk diffusion test on Mueller–Hinton (Bio-Rad, Marnes-La-Coquette, France) agar plates according to European Committee for Antimicrobial Susceptibility Testing (EUCAST 2023) recommendations (https://www.eucast.org/clinical_breakpoints accessed on 10 March 2023). Dalbavancin (DAL), ceftaroline (CPT) and ceftobiprole (CBP) MICs were determined by MIC Test Strips (Liofilchem, Roseto degli Abruzzi, Italy and BioMérieux, Marcy l’étoile, France). DAP, VAN and teicoplanin (TEI) MICs were determined using broth microdilution procedures (UMIC) (Bruker Daltonics, Champs sur Marne, France). Antibiotic susceptibility was interpreted using the EUCAST breakpoints.

### 4.2. Next-Generation Sequencing

The two pairs of isogenic (DAP-S/DAP-R) *S. aureus* strains were cultivated aerobically at 37 °C for 48 h on Columbia sheep blood agar plates (5%) (BioMérieux). Following the manufacturer’s instructions, genomic DNA (gDNA) was extracted from 200 μL of the overnight cultures suspension of the strains using DNeasy UltraClean Microbial Kit (Qiagen, Hilden, Germany) and eluted in 50 μL volume. Quality of gDNA was examined using Qubit fluorometer 2.0 (Invitrogen, Waltham, MA, USA). Whole Genome Sequencing (WGS) was carried out using Illumina MiSeq Sequencing system (Illumina, San Diego, CA, USA). Illumina library preparation was constructed using 250 ng of the extracted DNA following the Nextera XT DNA Prep Kit library paired-end protocol (paired-end read libraries, Illumina) and sequenced in a 39 h run providing 2 × 250 bp reads as previously described [38]. Quality control of the reads was performed directly on MiSeq output reads using FastQC software (v.0.11.7).

### 4.3. In Silico Analysis

After data quality validation, the *S. aureus* genomes were de novo assembled using Spades software (version 3.15.4) and blasted against the NCBI GenBank database, and also analysed on Type-Strain Genomes Server (https://tygs.dsmz.de/user_requests/new) online platform for more precision in bacteria identification. The first analysis of whole-genome MultiLocus Sequence Typing (wgMLST) was provided using EPISEQ^®^ CS V1-2 software (BioMerieux), a fully integrated web-based software application for genome assemblies and MLST. This software determined the percentage of sequence similarities between two genomes. The finalization of genome annotation was performed using CLC Genomics Workbench software (https://digitalinsights.qiagen.com/products-overview/discovery-insights-portfolio/analysis-and-visualization/qiagen-clc-workbench-premium/?cmpid=QDI_GA_DISC_CLC&gad_source=1&gclid=Cj0KCQiApOyqBhDlARIsAGfnyMqxFp3a-BExOSD0QM3ZwZXsO2JDw4EI5MinfGEm56EPCBijKUwYB_AaArP7EALw_wcB, accessed on 10 March 2023) (Qiagen, Germantown, MA, USA). Pangenome analysis was performed by comparison of the annotated *S. aureus* genomes using Roary tools (Version 3.13.0) available on Galaxy online software (https://www.usegalaxy.org.au/, accessed on 10 March 2023), then visualized on Phandango online tools. ResFinder 4.1, VirulenceFinder 2.0 and PlasmidFinder 2.1 were used for sequence analysis [39,40,41,42]. Antimicrobial resistance encoding genes, virulence, pathogenicity, and plasmids were in silico predicted using CGE online platform (http://www.genomicepidemiology.org/services/, accessed on 10 March 2023). Direct comparison between the strains isolated from the same patient was generated using BLAST Ring Image Generator (BRIG) [43]. Single-nucleotide polymorphisms (SNPs) analysis based on whole genome alignment was performed using Snippy [44]. SNP numbers between parental and antibiotic-exposed strains were interpreted according to the criteria of Ankrum and Hall [45], which defined strains with ≤71 SNPs as the “same” strains. All the identified genomics sequences have been deposited on the GenBank website accession bioproject: PRJNA1001284.

## 5. Conclusions

In conclusion, whilst DAP-R *S. aureus* have been previously described, few papers presented the in vivo acquisition of resistance. These two cases of MRSA isolates became resistant to DAP after around 3 weeks of exposure to this antibiotic. We suggest that *mprF* gene represents a hotspot target to acquire mutations to this antibiotic, particularly in MRSA. A new target has also been described on *cls* gene. This study highlights the non-systematic cross-resistance between DAP and glycopeptides. The use of DAP as first-line therapy at optimal dosages must be considered when patients are at risk of MRSA infection. Moreover, it is crucial to monitor DAP MIC in persistent MRSA bacteremia.

## Figures and Tables

**Table 1:** Main characteristics of the two pairs of isogenic (DAP-susceptible/DAP-resistant) *S. aureus* isolates.

Strain	Location	Collection Date	Specimen Type	ST	*spa* Type	MICs Values (mg/L):						Resistance Profile	% Sequence Similarities	Mutation in MprF and Cls Proteins *
						DAP	VAN	TEI	DAL	CPT	CBP			
A_DAP-S	Nîmes	26 April 2021	Blood	5	t777	0.5	1	0.5	0.06	0.25	1	PEN, OXA, OFX	99.96	-
A_DAP-R	Nîmes	16 May 2021	Blood	5	t777	2	1	1	0.06	0.38	1	PEN, OXA, OFX		L826F
B_DAP-S	Sète	3 September 2021	Blood	8	t622	0.5	1	0.5	0.06	0.38	1.5	PEN, OXA, OFX, RIF	99.93	-
B_DAP-R	Sète	26 September 2021	Blood	8	t622	2	1	0.5	0.06	0.38	1.5	PEN, OXA, OFX, RIF		S295L/*R228H*

CBP, ceftobiprole; CPT, ceftaroline; DAL, dalbavancin; DAP, daptomycin; TEI, teicoplanin; VAN, vancomycin.; PEN, Penicillin G, OXA, oxacillin, OFX, ofloxacin, RIF, rifampicin; * mutation in Cls protein is in italic.

## Data Availability

All the identified genomics sequences have been deposited on the GenBank website accession bioproject: PRJNA1001284.
